# OzBarley: A genetic and phenotypic data resource capturing the Australian barley breeding history

**DOI:** 10.1038/s41597-026-07056-y

**Published:** 2026-03-18

**Authors:** Ute Baumann, Elena Kalashyan, Julian Schwerdt, Amanda Box, Chris Brien, Kenneth Chalmers, Stewart Coventry, Aanandini Ganesalingam, Jessica Hyles, Brett Lobsey, Haoyu Lou, Sebastian Raubach, Sarah Richmond, George Sainsbury, Paul D. Shaw, Bjorg Sherman, John R. Stephen, Ben Trevaskis, Robbie Waugh, Matthew R. Tucker, Bettina Berger

**Affiliations:** 1 Adelaide University, Waite Research Institute and School of Agriculture, Food and Wine, Waite Campus, Urrbrae, SA 5064 Australia; 2 SECOBRA Recherches, Waite Campus, Waite Road, Urrbrae, SA 5064 Australia; 3 Australian Plant Phenomics Network, Adelaide University, Waite Campus, Urrbrae, SA 5064 Australia; 4 Australian Grain Technologies, 20 Leitch Road, Roseworthy, SA 5371 Australia; 5 InterGrain, 19 Ambitious Link, Bibra Lake, WA 6163 Australia; 6 CSIRO Agriculture and Food, GPO Box 1700, Canberra, ACT 2601 Australia; 7 Australian Grains Genebank, Grains Innovation Park, Horsham, Victoria 3400 Australia; 8 Information & Computational Sciences Department, The James Hutton Institute, DD25DA Invergowrie, Dundee, Scotland UK; 9 Bioplatforms Australia, Level 4, Building 4WW, Research Park Drive, Macquarie University, Macquarie, NSW 2019 Australia; 10 AGRF Ltd, Plant Genomics Centre, Waite Campus, Hartley Grove, Urrbrae, SA 5064 Australia; 11 Division of Plant Sciences, School of Life Sciences, University of Dundee, Dundee, DD1 4HN Scotland UK

**Keywords:** Plant breeding, Plant genetics

## Abstract

OzBarley is a comprehensive genotype-to-phenotype resource to support research and enhance barley breeding by integrating genotypic and phenotypic data for gene discovery. This publicly available dataset comprises genotypic data from historical and modern elite barley cultivars of significance to Australian barley breeding. The phenotypic component includes high-throughput imaging and X-ray CT-based spike analysis, enabling trait measurements such as plant growth dynamics and seed morphology. Users can leverage genome-wide association studies (GWAS) and genomic selection to identify genetic variants associated with agronomically important traits in the OzBarley datasets, thereby accelerating targeted breeding strategies. The dataset is accessible for download under CC-BY 4.0 license and users are invited to contribute new data when using OzBarley plant material in their research. Through its FAIR-compliant design (Findable, Accessible, Interoperable, Reusable), OzBarley represents a resource to protect genotypes of historical relevance, explore the genetic architecture of adaptation to dryland environments, and to enhance knowledge of the resilience, yield, and quality of barley cultivars under diverse environmental conditions, contributing to global food security and agricultural sustainability.

## Background & Summary

The advent of agriculture—around 12,000 years ago—marked the beginning of the Neolithic era and established the settled agrarian societies that reshaped human civilization. Cereal crops played a pivotal role during this time; their storable, calorie-rich grains ensured food surpluses, enabling the rise of specialised professions beyond subsistence and fostering cultural and technological innovation. Barley (*Hordeum vulgare*) was among the earliest cereals cultivated, with archaeological evidence for its domestication occurring in the Fertile Crescent of the Middle East circa 8000 BCE^1^.

Early agriculturalists’ selection of wild barley (*Hordeum vulgare subsp. spontaneum*) favoured a non-brittle rachis, preventing grain dispersal and allowing efficient harvest^2^. Traits such as spring growth habit, six-row spikes, and hull-less caryopses emerged later, improving cultivation and adaptability to diverse environments^3,4^. Despite a general narrowing of genetic diversity during domestication, barley landraces retained, lost or gained valuable traits, providing a reservoir of genetic diversity that remains relevant today.^5^

Building on landraces, from the late 19th and early 20^th^ century the selection and multiplication of genetically pure genotypes introduced the concept of ‘cultivars’ which were positively selected for growth, adaptability and end use quality. Subsequently, the introduction of systematic breeding techniques like hybridisation, mutagenesis and double haploidy opened the path to current genetic breeding technologies, such as marker-assisted and genomic selection, accelerating the development of cultivars with improved traits from disease resistance and abiotic stress tolerance to enhanced nutritional value^6,7^. For instance, the incorporation of genes conferring drought tolerance has been pivotal for cultivation in arid regions^8^. Even more recently, high-throughput RNA and DNA sequencing technologies have generated considerable amounts of sequence data that are becoming critical for both researchers and breeders^9^. Combined, these genetic and genomic resources enable the identification of quantitative trait loci (QTL) and candidate genes associated with desirable traits, to deliver targets which can potentially improve the efficiency and precision of breeding programs.

A significant challenge for breeding remains in the ability to effectively associate the wealth of genomic data available with phenotypic traits^10,11^. This problem arises from the complex interplay between genotype and the environment, which makes it challenging to predict phenotypes based solely on genetic information^12^. Traditional phenotyping methods are labour-intensive, time-consuming, and often lack the precision and throughput needed to capture complex traits controlled by multiple genes and environmental factors^13^.

The development of high-throughput phenotyping platforms can address these challenges, producing efficient and non-destructive measurement of a wide range of phenotypic traits under a range of conditions^14–16^. These platforms utilise advanced imaging systems, robotics, and data analytics to efficiently collect large volumes of phenotypic data^17,18^. When these experiments are designed in concert with high-throughput genotyping and transcriptome technologies, genetic markers and expression profiles can be efficiently linked with phenotypic traits. This integrated approach enhances the power of genome-wide association studies (GWAS), transcriptome-wide association studies (TWAS) and genomic selection models, facilitating the identification of candidate genes associated with desirable traits^19–22^.

These challenges and integrated approaches have special relevance in the Australian context. As the nation’s second-largest cereal crop, barley’s performance underpins significant economic output^23^. While previous studies report that Australian cultivars possess less genetic diversity than international sources^24^, they have been intensively selected to produce yield in the dryland environments where soil type, climate, and biotic stresses can differ significantly across regions^25,26^. As such, they are a crucial genetic resource for understanding the complex traits needed to address the compounding demands of modern breeding — from mitigating climate change and abiotic stresses to ensuring yield security, disease resistance, and end-use market quality^27–31^.

In this manuscript, we present OzBarley, a genotype-to-phenotype data asset of historic and modern Australian barley cultivars, international founder lines and landraces that aims to bridge the gap between high throughput ‘omic data and practical breeding applications. This is a publicly available, FAIR-compliant (findable, accessible, interoperable and reusable)^32^ resource designed for the research and breeding community. The primary data asset comprises genotypic variation data and transcriptome data —generated with Illumina XT genotyping and RNA-Seq experiments—for the OzBarley elite panel of over 200 barley accessions selected from the Australian barley breeding pedigree. These data are integrated with high-throughput phenotypic data that enables plant growth modelling and longitudinal trait measurements. By combining extensive genotypic profiles and transcriptome data with detailed phenotypic observations, OzBarley provides user with a powerful data asset for identifying genotype-to-phenotype associations via GWAS and TWAS approaches. The addition of Illumina XT SNP data for a panel of 500 barley lines, comprised of predominantly landraces, some international cultivars and breeding lines (OzBarley landrace panel) further enables allele mining for traits of interest.

Openly accessible for download under a CC-BY- 4.0 license, users can access the dataset to explore gene-trait relationships presented in the dataset, allowing breeders and researchers to draw novel correlations. Users are invited to explore a range of phenotypic traits and to contribute novel phenotypic data in the future which can be used to enhance selection efficiency, and develop improved barley cultivars adapted to specific environmental conditions and market demands. OzBarley seed will become available via the Australian Grains Genebank.

## Methods

### Genotype selection

Genotypes included in OzBarley elite panel were selected based on their relevance to Australia’s barley breeding history. These were identified through engagement with current and former public and private breeding programs, the broader barley research community, and the Australian Grains Genebank. The final OzBarley elite panel represents the genetic diversity and agronomic relevance of Australian barley germplasm, and encompasses international founder lines, historical varieties, modern commercial cultivars, advanced breeding lines and some landraces. Selection focused on cultivars with significant contributions to yield, quality and stress tolerance. Additionally, cultivars with traits favored by the brewing, feed and food industries were included to ensure market relevance.

**Fig. 1:**
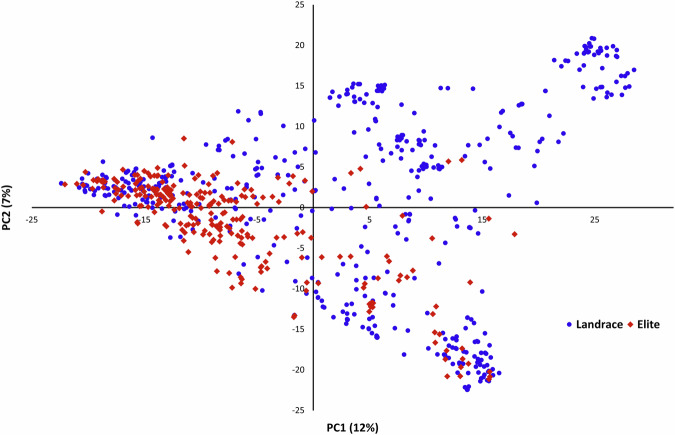
Principal component analysis (PCA) of barley landrace and elite lines based on the XT chip genotype data shows the diversity among genotypes. PC1 (x-axis) contributes 12% of the total variation, PC2 (y-axis) contributes 7%.

**Fig. 2:**
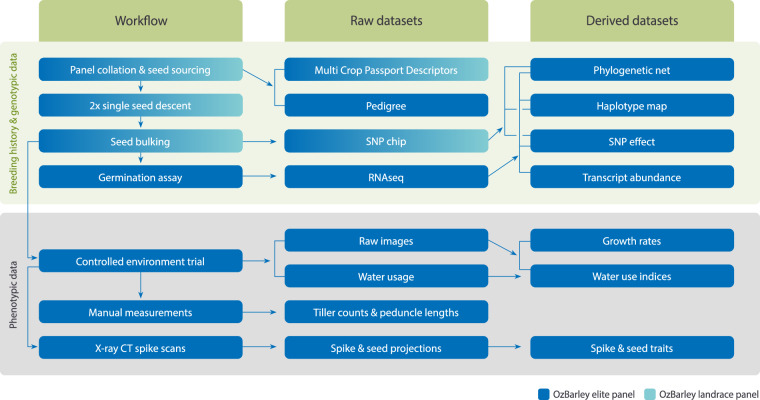
Flowchart detailing the operations to curate the OzBarley panels and obtain genotypic and phenotypic data (left column). The data collected at the various stages (middle column) or derived from primary data (right column) is detailed and datasets are available for download from the OzBarley Figshare collection.

OzBarley landraces were selected from the Australian Grains Genebank catalogue using stringent criteria. Each genotype was required to fall within the lowest third for plant height, the earliest third for maturity, and represent a balanced distribution of 2-row and 6-row types across diverse geographic origins. After two rounds of single-seed descent, 500 landraces were included in the OzBarley landrace panel. The genetic diversity among these landraces, along with elite barley lines, is illustrated in a principal component analysis (PCA; Fig. 1), showing both overlap and genetic distinctions between the groups. The workflow from panel assembly to data acquisition is detailed in Fig. 2 and below.

### Plant material

The majority of the OzBarley genotypes were supplied by the Australian Grains Genebank (Horsham, Australia). Others were generously supplied by colleagues from breeding companies and university seed stores. Meta data was collated for an original list of 300 elite genotypes, with 222 lines remaining after two rounds of single-seed descent, with resulting genotypic and phenotypic data for those lines. Approximately 15% of the OzBarley elite panel is 6-row, the majority is 2-row cultivars.

### Metadata

The metadata for each OzBarley accession (elite and landrace panel) was collated to fulfill Multi-Crop Passport Data (MCPD) record requirements. Data were sourced from Genesys^33^, the U.S. Department of Agriculture Germplasm Resources Information Network^34^, metadata provided by the Australian Grains Genebank (AGG) and breeding literature. All collected metadata were standardised according to the MCPD V.2.1 guidelines^35^ and are available for download (see Data Records). Pedigree information for the OzBarley elite panel was similarly collected from Genesys, AGG, USDA-GRIN databases, breeding literature, and a local copy of the International Crop Information System database^36^. Pedigrees were encoded using the three-column and Purdy notation formats^37^. Controlled vocabularies and standardised units were used to promote consistency and facilitate data integration.

### Plant growth

All OzBarley elite panel lines and OzBarley landrace panel lines underwent two rounds of single-seed descent in a greenhouse environment under long-day conditions for accelerated growth (Australian Grain Technologies, Roseworthy). After completion of the single-seed descent, seed was used for SNP genotyping (see below). For lines that had little seed after single-seed descent, leaf material was used for SNP genotyping instead. Six seeds were planted across two 4.5 L pots per line in a standard coco-peat potting mix for seed increase. Plants were grown in a controlled-environment greenhouse with a 22 °C/15 °C day/night temperature cycle and natural lighting between November 2021 and March 2022 (OzBarley elite panel) and between June 2023 and February 2024 (OzBarley landrace panel) at the Plant Accelerator, Australian Plant Phenomics Network (APPN), Adelaide University. At maturity, three spikes per OzBarley elite line were harvested for X-ray computed tomography (CT) analysis (see below). The remainder of the spikes were harvested and threshed for subsequent use (i.e. additional bulking for depositing into the Australian Grains Genebank, controlled environment phenotyping, seed multiplication in the field).

### X-ray CT imaging and analysis

X-ray CT measurements of OzBarley elite panel spikes were conducted at The Plant Accelerator. The barley spikes were individually labelled and placed in customized sample holders. Each sample holder accommodated 30 barley spikes in a vertical arrangement, enabling simultaneous scanning using an X-ray CT scanner (Phenokey, ‘s-Gravenzande, The Netherlands; Fraunhofer Institute of Integrated Circuits, Erlangen, Germany). The scans were performed at 220 kV voltage and 6 mA current, utilizing a 0.5 mm copper filter for image acquisition at a resolution of 84 µm. The specimen stage rotated 220 degrees with a rotation step increment of 0.15 degrees. Throughout the 7-minute scanning duration, 1500 projections were collected at an exposure time of 250 ms each. Automated correction and reconstruction were carried out using the Fraunhofer EZRT CTProcessingNet, following the approach described by Schmidt *et al*.^38^. The resulting reconstructed 3D grey-scale volume, with a depth of 16 bits, was then segmented into 30 individual 3D volumes using the EarSTubesSegmentation algorithm (Fraunhofer Institute of Integrated Circuits, Erlangen, Germany), with each volume file representing a single spike. For each spike, automatic seed segmentation and measurements were conducted using the EarS algorithm to determine various traits, including mean attenuation value, spherical ratio, spatial position in x, y, z coordinates, size in voxel, surface area in voxel, and virtual weight^38^.

Based on the raw data obtained from the EarS algorithm, the following traits were analyzed at the individual seed level: seed length, estimated seed weight; sphericity ratio (values between 1 and 0, where 1 indicates a perfect sphere, 0 an infinite line); seed volume; seed surface area; and spike level: seed count; total seed weight per spike; spike length.

### High-throughput controlled-environment phenotyping experiment

The experiment with the OzBarley elite panel was conducted in the two southern Smarthouses using the conveyor system of a LemnaTec Scanalyzer 3D (LemnaTec GmbH, Aachen, Germany) at the Australian Plant Phenomics Network facility at Adelaide University. The experiment involved 1110 carts, each with a single plant, and the experimental area consisted of 20 Lanes × 26 Positions on the conveyor system in the SW Smarthouse and 24 Lanes × 26 Positions on the conveyor system in the SE Smarthouse, although not every position in a lane is occupied by a pot with a plant. The experiment was designed for 222 genotypes and the experimental area was divided into five irregular Blocks of 222 carts, with two blocks in each Smarthouse plus one block split between the Smarthouses. Each Block containing one replicate of the 222 genotypes. To maximize statistical precision, each Block was divided into a semi-regular grid of subBlocks such that each Block comprised 14–16 subBlocks that ranged in size from 2 × 5 to 4 × 5 carts. The R package odw^39^ was used to produce an optimal allocation of the genotypes to the subBlocks and the R package dae^40^ was used to randomize the design. The design is illustrated in *Experimental design and growth curve figures* (Figs. 1–4, see Data Records).

Planting occurred on 7 April 2022, denoted day after planting (DAP) 0. Three plants died during the experiment, while one was deemed to be an outlier. All four were excluded from analysis. The analysis data set thus comprised 1106 plants. Further, the results of genotyping the panel from which the plants in this experiment were selected, carried out after the experiment, revealed that there were only 215 distinct genotypes involved.

### Phenotypic data preparation

#### Projected shoot area data

Imaging was carried out daily from DAP 15 to DAP 69 inclusive, and thence every second day until DAP 116, the SE Smarthouse being imaged on odd-numbered DAPs and the SW Smarthouse on even-numbered DAPs. All plants were watered to weight daily throughout the experiment, with twice-daily watering over the period DAP 48–69.

Based on the images obtained using RGB cameras, the Projected Shoot Area (PSA kilopixels) of each plant was calculated as the sum of the number of plant pixels from three camera views: two side views and a view from above. The imaging data was prepared using the SET method described by Brien *et al*.^41^. Computations were carried out using the R package growthPheno^42^. The absolute PSA growth rates (PSA AGR, kpixels per day) and the relative PSA growth rates (PSA RGR per day) were calculated by differencing consecutive PSA and ln (PSA) values, respectively, and dividing by the time differences. Data cleaning was carried out by smoothing the PSA for each plant to remove the systematic biases arising from transitory events and affecting the growth trend and rates for single DAPs. The smoothed PSA (sPSA kpixels) for each plant was produced using the traitSmooth function from **growthPheno** to smooth the logarithms of its PSA using cubic P-splines with a smoothing penalty of 31.6. The sPSA AGR and sPSA RGR were then calculated by differencing as for the PSA (*Experimental design and growth curve figures*, Figures 5–7, see Data Records).

#### Height data

The height of each plant was estimated from the two-side view RGB images used in the PSA analysis. For each plant and imaging day, the apparent height (pixels) was computed for each side view. The rescaled maximum of these two values (1 cm = 31 pixels) was taken as the Height of the plant.

#### Water data

Pot weights before and after watering were available for each imaging DAP. Daily water use (WUR mL per day) was estimated based on net change in pot weight between successive imaging days. No attempt was made to differentiate between biological consumption and evaporative loss. The process for smoothing the PSA was applied to the WUR to produce the smoothed WUR (sWUR mL) per day by smoothing the WUR using cubic P-splines with a smoothing penalty of 31.6. The smoothed Water Use Index (sWUI kpixels/mL) is calculated as the ratio of the sPSA AGR to the sWUR (*Experimental design and growth curve figures*, Figures 8–9, see Data Records).

#### Tiller data

For each plant, the number of tillers was counted manually on two occasions, for a total of three traits:

- Early Tillers (DAP 41)
- Late Tillers (DAP 117–119)
- Tiller Increase (difference between Late Tillers and Early Tillers).

Subsequently, two single-tiller traits were measured as follows:

- Tiller Height (cm)
- Peduncle Length (cm)

Wherever possible, three (flowering) tillers were sampled per plant and then assessed for both traits. The potential number of plants in this study was *n* = 4 × 222 = 888, since one Block of 222 plants was excluded. The actual number of plants with at least one recorded tiller measurement was 857 (Tiller Height) and 854 (Peduncle).

#### Growth data

Growth was investigated by dividing the imaging period into eight intervals: DAPs 15–27, 27–42, 42–60, 60–72, 72–79, 79–91, 91–103, and 103–115. A total of 45 traits were computed:

- **single-day responses:** sPSA for DAP 15, 27, 42, 60, 72, 79, 91, 103 and 115
- **interval responses:** sPSA AGR, sPSA RGR, sWUR and sWUI for each of the DAP intervals 15–27, 27–42, 42–60, 60–72, 72–79, 79–91, 91–103 and 103–115; and
- **single responses:** Maximum sHt, Time (DAP) of Maximum sHt, Maximum sWUR, Time (DAP) of Maximum sWUR.

Each of these traits yields a single value for each plant.

### Statistical analysis of the phenotypic data

To produce phenotypic best linear unbiased estimates (BLUEs) for the genotypes, each trait was analyzed using the R packages ASReml-R^43^ and asremlPlus^44^. The following maximal linear mixed model was fitted to each trait:

$${\bf{y}}={\bf{1}}\mu +{{\bf{X}}}_{{\rm{t}}}{\boldsymbol{\tau }}+{{\bf{X}}}_{{\rm{s}}}{\boldsymbol{\beta }}+{{\bf{Z}}}_{{\rm{d}}}{{\bf{u}}}_{{\rm{d}}}+{{\bf{Z}}}_{{\mathcal{l}}}{{\bf{u}}}_{{\mathcal{l}}}+{\bf{e}},$$

where ${\bf{y}}$ is the response vector of values for the trait being analyzed; $\mu$ is the overall mean in this experiment for the response; ${\boldsymbol{\tau }}$ is the vector of fixed effects of interest; ${\boldsymbol{\beta }}$ is the vector for fixed large-scale spatial effects; ${{\bf{u}}}_{\mathrm{d}}$ is the vector of random large-scale spatial effects arising from the design used; ${{\bf{u}}}_{{\mathcal{l}}}$ is the vector of random coefficients for the terms used in modelling the local spatial effects; ${\bf{e}}$ is the vector of residual effects. The vector ${\bf{1}}$ is the vector of ones; the matrices ${{\bf{X}}}_{t}$, ${{\bf{X}}}_{\mathrm{B}}$, and ${{\bf{Z}}}_{\mathrm{d}}$ are the design matrices for the corresponding effects; ${{\bf{Z}}}_{{\mathcal{l}}}$ is the vector of random effects for the tensor-product splines or for an autocorrelation model of order one used in modelling the local spatial variation.

The vector ${\boldsymbol{\beta }}$ of fixed spatial effects consists of just the single vector ${{\boldsymbol{\beta }}}_{\mathrm{S}}$ for the Smarthouse (S) effect that allows for an overall difference between the two Smarthouse. Similarly, the vector ${\boldsymbol{\tau }}$ of fixed effects of interest consists of the single vector ${{\boldsymbol{\beta }}}_{\mathrm{G}}$ for the Genotype effects. The vector ${{\bf{u}}}_{\mathrm{d}}^{{\rm{\top }}}$ is partitioned into two subvectors, $$[\begin{array}{c}{{\bf{u}}}_{{{\rm{d}}}_{{\rm{W}}}}^{{\rm{\top }}}{{\bf{u}}}_{{{\rm{d}}}_{{\rm{E}}}}^{{\rm{\top }}}\end{array}]$$, where the subvectors contain the random large-scale spatial effects for Smarthouse SW and SE, respectively. Each of these subvectors is further subdivided into five subvectors, with ${{\bf{u}}}_{{\mathrm{d}}_{\mathrm{W}}}^{{\rm{\top }}}$ subdivided as follows: $$[\begin{array}{c}{{\bf{u}}}_{{\mathrm{B}}_{\mathrm{W}}}^{{\rm{\top }}}{{\bf{u}}}_{{\mathrm{B}:\mathrm{R}}_{\mathrm{W}}}^{{\rm{\top }}}{{\bf{u}}}_{{\mathrm{C}}_{\mathrm{W}}}^{{\rm{\top }}}{{\bf{u}}}_{{\mathrm{B}:\mathrm{C}}_{\mathrm{W}}}^{{\rm{\top }}}{{\bf{u}}}_{{\mathrm{B}:\mathrm{R}:\mathrm{C}}_{\mathrm{W}}}^{{\rm{\top }}}\end{array}]$$, and ${{\bf{u}}}_{{\mathrm{d}}_{\mathrm{E}}}^{{\rm{\top }}}$ subdivided analogously. The five subvectors allow for random variation between (i) the Blocks in Smarthouse, (ii) Rows within Smarthouse Block, a Row consisting of between 2 and 5 Lanes, (iii) Columns within a Smarthouse, consisting of 5 or 6 Positions, (iv) Columns within a Smarthouse Block, and (v) the combinations of the Rows and Columns within a Smarthouse Block.

The residual effects, ${\bf{e}},$ reflect the random variation between individual plants in the experiment and they were assumed to be normally distributed with population mean equal to zero and two variances that differed between the Smarthouses.

To allow for local spatial variation, four models for the local spatial variation were fitted and compared for each trait: (i) autocorrelation of order one, (ii) natural cubic smoothing splines, (iii) cubic P-splines, and (iv) no local spatial variation. Each model involved separate submodels for each Smarthouse. The model with the smallest Akaike Information Criteria (AICs) was declared the best fitting model. Ultimately, given that cubic P-spline models either had or were close to the minimum AIC for all traits, the local spatial variation was always modelled using them. For each trait, BLUEs were produced along with their standard errors.

### Genetic analysis

#### Genotyping

Genomic DNA was extracted from 6 bulked seeds per accession using a sbeadex kit (LGC Genomics, Hoddesdon, UK), and from leaf tissue harvested from individual accessions using a MagMax™ Plant DNA Isolation Kit (Thermo Fisher Scientific, Waltham, USA). Genotyping was conducted using the Infinium™ Wheat Barley 40 K v1.0 BeadChip (Elite Panel) and v1.1 BeadChip (Landraces) (Illumina, San Diego, USA)^45^.

DNA extraction and interrogation of the chip is described by Azizinia *et al*.^46^.

#### RNA Sequencing of OzBarley elite panel

Eight seeds per line were placed in a petri dish containing two Whatman filter papers (size #1) and 4 ml sterile H_2_0. Plates were sealed with parafilm, covered in foil in single layers (10 plates per foil packet) and placed in a growth cabinet (Conviron Australia, A1000). The cabinet was set to a constant 20 °C without lighting. A temperature monitor (Kestrel drop D1) logging data every 30 minutes indicated that actual temperatures fluctuated between 19.5 to 22.9 °C. After 4 days growth, seedlings were harvested by removal of the endosperm and collection of roots, embryo and coleoptile into a 2 ml tube containing 2 stainless steel ball bearings and snap freezing in liquid nitrogen. Two separate seedlings were harvested, one for RNA extraction and a second backup sample (stored at −80 °C). A single sample per line was used for RNA sequencing.

RNA was extracted from samples using a Maxwell ® RSC Plant automated extraction system (48 samples per run, 5 batches in total). Manufacturer’s instructions were followed (Promega, catalogue number AS1500) with the following modifications. Qiagen Tissue Lyser (II) carrier plates were placed at −80 °C for 2 hours to chill, samples removed from −80 °C and placed on dry ice before loading into tissue lyser. Tube grinding occurred at a frequency of 30/sec for 1 minute, then samples were re-orientated by turning the plate and repeating for an additional minute before samples were returned to dry ice. A total 450 µl of homogenising buffer (Thioglycerol 20 µl/ml) was added to samples, vortexed and tubes left on wet ice while remaining samples were processed. Then 225 µl lysis buffer was added, samples vortexed and left at room temperature for 10 minutes. Tubes were centrifuged at 12,000 rpm for 2 minutes in an Eppendorf benchtop microfuge (catalogue number 5415D) and 600 µl of supernatant added to the Maxwell ® cassette. DNase was added (5 µl) and samples eluted into 55 µl H_2_0.

RNA quality was checked by agarose electrophoresis and quantified using a NanoDrop spectrophotometer (catalogue number ND-ONE, Thermofisher Scientific). RNA was then subject to library development and sequencing.

Library preparation and RNA sequencing was carried out at the Australian Genome Research Facility (AGRF). Libraries were constructed from 300 ng total RNA (RIN > 7.0) using the Illumina TruSeq Stranded mRNA Library Prep kit. Sequencing was performed using the Illumina NovaSeq 6000 at 20 million reads per sample, using 150 nucleotide paired-end reads.

#### RNASeq processing

OzBarley uses the Barley cv. MorexV3^47^ genome assembly as the reference genome, downloaded from GrainGenes (https://wheat.pw.usda.gov/GG3/content/morex-v3-files-2021).

RNAseq data (Illumina) for 222 elite lines were processed including adapter and quality trimming by fastp v0.19.7^48^ using the following parameters:

--detect_adapter_for_pe --length_required = 100 --trim_front1 = 10 --cut_tail -W 1 -M 26 --trim_poly_g --trim_poly_x --qualified_quality_phred 28 --n_base_limit = 4.

The quality of raw data and trimmed reads were examined by FastQC v0.11.5 (https://www.bioinformatics.babraham.ac.uk/projects/fastqc/), MultiQC v1.7^49^ and fastp v0.19.7^48^ The resulting reads were aligned to the reference genome using STAR v2.7.3a^50^ using the following parameters:

--outFilterMismatchNoverLmax 0.04 \

--outFilterMultimapNmax 1 --outFilterMultimapScoreRange 0 --outFilterScoreMinOverLread 0 \

–alignEndsType Local --alignIntronMax 12000 --alignMatesGapMax 13500\

--alignSoftClipAtReferenceEnds No \

--outSJfilterOverhangMin 35 20 20 20 \

--outSJfilterCountTotalMin 10 3 3 3 \

--outSJfilterCountUniqueMin 5 1 1 1 \

--outSAMtype BAM SortedByCoordinate --limitBAMsortRAM 160000000000\

--outSAMstrandField intronMotif --outSAMattributes All \

The unique mapping rates were in a range of 75-93% across the samples, with 1.5-3% of reads multi mapped and 6-23% failed to align.

RNA-seq expression was determined by using in-house R script (R v3.5.1), utilising Rsubread v1.32.4^51^ and edgeR v3.24.3^52^ libraries. We computed counts per million (CPM) and provide data in two file formats.

#### Variant calling

For GWAS analysis we performed a stringent variant calling using BCFtools v1.9^53^ with the following parameters:

bcftools mpileup–annotate FORMAT/AD,FORMAT/DP --min-BQ 25 --min-MQ 25 --rf 2

bcftools call–multiallelic-caller --variants-only

bcftools norm

bcftools filter --soft-filter FAIL --exclude ‘FORMAT/DP < 5’

bcftools filter --regions-file “exons from Hv_Morex.pgsb.Jul2020.exons.bed”

bcftools filter --exclude ‘FILTER = “FAIL”’

bcftools filter --exclude ‘%QUAL < 50’

bcftools filter --exclude ‘INFO/INDEL = 1’

For general variant calling we used the same BCFtools v1.9 pipeline without filtering and additionally VCFtools v0.1.15^54^ bcftools mpileup --annotate FORMAT/AD,FORMAT/DP --min-BQ 25 --min-MQ 25 --rf 2

bcftools call --multiallelic-caller --variants-only

bcftools norm

bcftools filter --regions-file “exons from Hv_Morex.pgsb.Jul2020.exons.bed”

vcftools --gzvcf --minDP 3 --recode --recode-INFO MQ --recode-INFO BQB -c \

Variants that resulted from this were further analysed by SnpEff v4.3i^55^ with the parameters -no-intergenic MorexV3.

## Data Records

OzBarley is open without any restrictions or embargo and data are formatted using widely accepted standards and open formats.

The OzBarley data asset is accessible via a central OzBarley Figshare Collection^56^. From this Collection, data can be accessed via three associated Datasets described in more detail below. Files are either available for download, or hyperlinks are provided for externally hosted files, where file number of file size was not suitable for Figshare deposit. A dynamic view of the OzBarley data asset is also hosted on the Germinate platform^57^, accessible at https://ozbarley.plantphenomics.org.au/.

### OzBarley phenotypic data

The OzBarley phenotypic data is available on Figshare^58^.

#### OzBarley time course image data-hyperlinks

The excel file contains a readme sheet describing the column headers of the main data sheet. In brief, OzBarley accession names and OzBarley IDs are provided for each plant grown during the controlled environment growth experiment using a LemnaTec 3D Scanalyzer. In addition, the Smarthouse location and replicate of each plant is provided. Hyperlinks to the raw images collected during the experiment are provided for each plant, thus multiple replicate entries per OzBarley accession. Each hyperlink resolves to a landing page where time course zip folders can be downloaded for single images from two fluorescence cameras (side view – SV; top view – TV) and for 19 red-green-blue images from four cameras and six rotational angles (0 to 5, at 20° rotation).

#### OzBarley_imaging_tiller_data_BLUEs

The excel file contains multiple sheets providing the data derived from the raw images and manual measurements of plants grown during the controlled environment experiment. A Genotype Key provides the required metadata to map the OzBarley accession (Genotype) with its corresponding OzBarley ID. A second sheet, Trait terms imaging, describes the phenotypic traits derived from the analysed images with full vocabulary names, abbreviated column headers used elsewhere in the file, description, data type and units. The sheet BLUEs imaging provides the BLUEs and standard errors calculated for the individual phenotypic traits derived from image data as described in the Methods section. Trait term tillers provides the manually collected phenotypic traits for tillers and peduncles, following the same format as the imaging trait terms. The final sheet contains the BLUEs and standard errors for the tiller and peduncle traits.

#### Experimental design and growth curve figures

The word document contains figures, detailing the experimental design of the controlled environment growth experiment and smoothed growth curves extracted from the time course image data and relates to the method section of this manuscript.

#### Timelapse_Video_Dictator_MaximusCL_Unicorn_Vlamingh

An MP4 timelapse video provides an example of OzBarley accessions Dictator, Maximus CL, Unicorn and Vlamingh growing during the course of the controlled environment experiment.

#### OzBarley X-ray Image metadata, and Spikes and Grains zip folders

The excel file for the X-ray image metadata should be used in conjunction with the two zip folders for spike and grain images. Spikes harvested from greenhouse-grown OzBarley plants were imaged with an X-ray CT scanner for non-destructive spike and grain phenotyping. The metadata file provides a readme sheet to explain column headers of the metadata sheet. In brief, details and metadata for each png image file in the two associated zip folders is provided. In the Spike zip folder, for each OzBarley accession, one spike is presented by the maximum intensity projection view of the scanned spike specimen, constructed by projecting the voxel with highest attenuation value through the 3D volume onto a 2D image. For each of those spikes, a png file in the Grains folder presents all segmented grains at the maximum transverse section area.

#### OzBarley_X-ray_raw_data

The excel file contains the numeric data extracted from the X-ray CT spike scans, with three spikes scanned for each OzBarely accession. The Trait Description sheet provides information on the column headers of the two data sheets. Numeric data is provided for the total spikes, such as spike length and seed number per spike. The seed data is presented with an individual data row for each seed identified, thus multiple data rows per scanned spike.

### OzBarley genotypic and expression data

#### Raw RNA sequencing data

Raw data are deposited in the NCBI Sequence Read Archive under accession number PRJNA1152269 (SRP529507)^59^^,^^60^. The data are provided in FASTQ format.

#### 2025_RNASeqSNP_and_Genotype data and readme – HapMap

The readme file in rtf format provides important notes on the OzBarley elite panel SNP data and its usage. The OzBarley elite panel was genotyped using the Infinium™ Wheat Barley 40 K v1.0 BeadChip (Illumina, San Diego, USA). In addition, RNA sequencing of the OzBarley elite panel was conducted using 4-day old seedlings (see Methods section). The SNP data derived from both data sources was aligned to Barley cv MorexV3 and the data is provided in HapMap format (*2025_RNAseqSNP_and_GenoData.hapmap*).

#### OzBarley landrace panel SNPchip data

The OzBarley landrace panel was genotyped using the Infinium™ Wheat Barley 40 K v1.1 BeadChip (Illumina, San Diego, USA). Marker positions are based on the Barley cv Morex V3 assembly^46^. OzBarley IDs and line names are provided as concatenated identifier in the first column.

#### snp_description and readme – snp_description

The readme file in rtf format provides important information on data reuse. SNPs identified from the RNA-Seq data were combined with genotype calls obtained using the Illumina XT Wheat-Barley SNP chip. The *snp_description* CSV file provides information on the genomic location of each SNP and their predicted effects on genes. Variant effects were determined using the SnpEff software (https://pcingola.github.io/SnpEff/).

#### cpm_count and readme – cpm_count

Gene expression data was obtained via RNAseq from germinating OzBarley seedlings. The readme file in rtf format contains important information for data reusage. In the two transcript abundance files (*cpm_counts.tsv* and *cpm_count.xlsx*) the read counts are expressed in CPM (counts per million reads). The data was aligned to Barley cv MorexV3. The final column in the CPM files provides gene annotations, which were obtained alongside the Barley cv MorexV3 genome assembly (version Hv_Morex.pgsb.Jul2020) from GrainGenes (https://wheat.pw.usda.gov/GG3/).

SNP effect and expression data can also be explored with a user interface at https://ozbarley-explorer.plantphenomics.org.au/.

#### OzBarley pedigree_2025_final3c and OzBarley pedigree

The OzBarley elite panel pedigree is provided in txt format for import and display in Helium software (https://github.com/cardinalb/helium-docs/wiki). The data was collated from multiple sources as described in the Methods section. A graphic representation of the pedigree is available as png file (*OzBarley pedigree*), where OzBarley elite lines are presented as blue octagons, other lines required for displaying the pedigree, but not part of the OzBarley panel, are presented as black circles. The symbol size represents the relative contribution to the pedigree.

#### OzBarley Phylogenetic Network and OzBarley phylogenetic network metadata

A phylogenetic network analysis of OzBarley SNP data was performed using PLINK and NetViewR. The pdf file provides a graphic representation of the network. The associated excel metadata file contains the Node IDs (OzBarley IDs) and OzBarley accession names. Two sheets are provided, one in standard tabular format, the second has been edited for easier printing and viewing alongside the pdf figure.

#### OzBarley Multi-Crop Passport Descriptors

OzBarley lines were described according to the Multi-Crop Passport Descriptor (MCPD) convention (https://hdl.handle.net/10568/105205). Data is provided in Excel format and can also be accessed on the OzBarley Germinate instance https://ozbarley.plantphenomics.org.au/). Not all OzBarley elite and landrace lines with entries in this file are represented in the genotypic and phenotypic datasets.

### OzBarley raw image data

While the raw images are externally hosted due to the large file number, the Figshare dataset provides an additional link the *OzBarley Time Course Image Data-Hyperlinks* file (under Related materials)^61^. This excel file provides the necessary metadata to associate the image data listed with barcode numbers and the respective plant grown during the controlled environment growth experiment. For each plant/barcode, 21 zip folders are provided. Two zip folders for Fluorescent images from a top view and sideview camera (*FLUO_TV* and *FLUO_SV*), one zip folder for red-green-blue (RGB) images from a top view camera (*RGB_3D_3D_top*) and 18 zip folders from three RGB cameras each (*RGB_3D_3D_side_upper*, *RGB_3D_3D_side_lower*, *RGB_3D_3D_side_lower*) at six rotational angles each (*0* to *5*, at 20° rotation each).

## Technical Validation

### Metadata and pedigree

Missing and conflicting entries existed for both metadata and pedigree information. Careful manual curation was required and involved cross-referencing among academic references, breeding literature and online databases: genesys (https://www.genesys-pgr.org); USDA-Grin (https://www.ars-grin.gov) and plant breeder rights searches (https://ipsearch.ipaustralia.gov.au/pbr/). Additionally, direct communication with barley breeders and genebank curators, provided important confirmation. All metadata were standardized according to MCPD V.2.1 guidelines (FAO/Bioversity, 2017). A visual representation of the pedigree is available online^59^ and shows the pedigree of OzBarley cultivars, visualised in Helium^62^ along with documented parents outside the panel.

### Seed lots

Seed lots were assessed for homogeneity and specific phenotypic traits, such as the presence or absence of hulls or aleurone colour, through visual inspection. To assess whether each seed lot matched its accession metadata, phenotypic assessments were cross-referenced with SNP data and pedigree records. Visual inspection of spikes was employed to examine grain row structures, awned versus hooded seed, providing additional confirmation of phenotypic traits aligned with the accession metadata collected from genebank and historical records. Shoot morphology of plants was observed during greenhouse experiments to compare to known growth habits of key cultivars (*Timelapse_Video_Dictator_MaximusCL_Unicorn_Vlamingh*^59^). For some lines, SNP genotyping was repeated to confirm results and compared to data from different seed sources.

### Genotype quality control using SNP data

SNP positions identified in the RNAseq data and the Infinium™ Wheat Barley 40 K v1.1 BeadChip (Illumina, San Diego, USA) were carefully curated to identify overlapping SNP loci, with over 1,200 common SNPs between the two datasets. The overlapping SNPs were used to ensure genotyping samples and seed material were not mixed up.

### Phenotypic data preparation

Imaging data were processed using the SET method described by Brien *et al*.^41^, and computations were performed with the R package **growthPheno** (Brien, 2023)^42^, facilitating reproducible analysis. Exploratory smoothing of traits (*Experimental design and growth curve figures*^58^, Figures 5–9) was carried out using the traitSmooth function from **growthPheno** to compare the fits of different types of splines and settings of the smoothing parameters methods. The type and parameter setting judged to have the superior fit was selected for smoothing the PSA and WUR. This approach cleans the data by smoothing the PSA and WUR for each plant to remove the systematic biases arising from transitory events and affecting the growth trend and rates for single DAPs.

For tiller data, manual counting was performed on two occasions, and measurements of tiller height and peduncle length were taken from up to three flowering tillers per plant.

### Statistical analysis of the phenotypic data

Residual-versus-fitted values plots and normal probability plots of the residuals for each trait were inspected to check that the assumptions underlying their analyses were met. The residual plots for all traits were satisfactory, indicating that the selected models appear to be appropriate.

## Usage Notes

The OzBarley data is available for download at the OzBarley Figshare Collection^56^ and its associated datasets^58,59,61^ in a version-controlled repository. The OzBarley data asset is also accessible in a dynamic view for data exploration through the Germinate platform, available at https://ozbarley.plantphenomics.org.au/. The combination of phenotypic and genotypic data available to users allows Genome-wide association studies to be performed.

## Data Availability

RNAseq data is available at the NCBI Sequence Read Archive under accession number PRJNA1152269 (SRP529507)^60^. Image data, genotypic and phenotypic data is available for download on the Adelaide University Figshare repository. All datasets are linked to a single Figshare collection^56^ (10.25909/c.6952545).

## Code availability

No custom code was used.
